# Supplementary material for: The interactome of intact mitochondria by cross-linking mass spectrometry provides evidence for coexisting respiratory supercomplexes
Source: Mol Cell Proteomics. 2017 Dec 8;17(2):216–32. doi: 10.1074/mcp.RA117.000470 (PMC5795388; doi:10.1074/mcp.RA117.000470)
Supplement: Supplemental Data [file supp_RA117.000470_133922_0_supp_23978_fzffwf.zip › spectra_annotation/mito_DR_spectra_annotation/498-1-5-1-3-1.pdf]

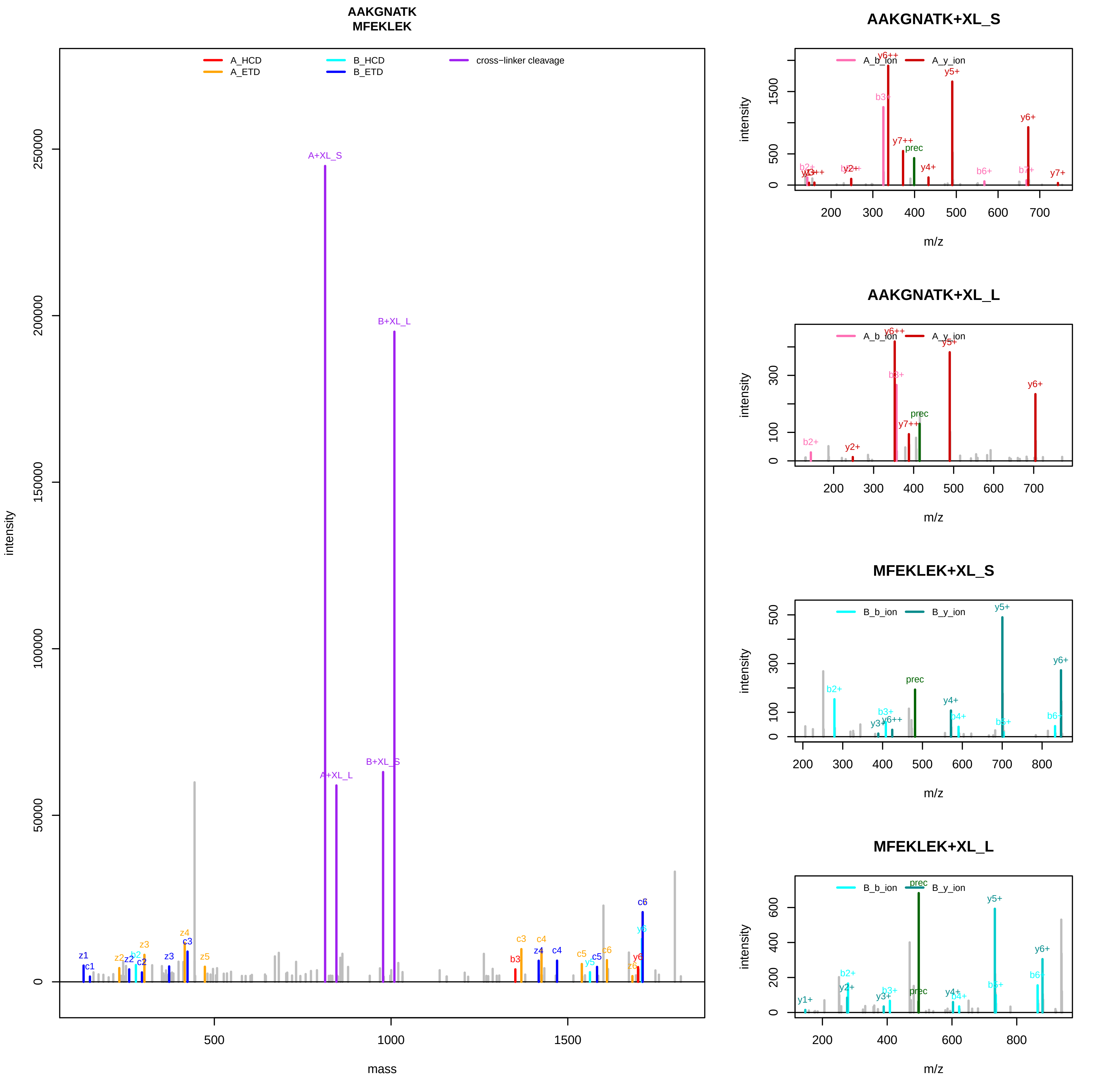

AAKGNATK
MFEKLEK
A_HCD
A_ETD
B_HCD
B_ETD
cross−linker cleavage
A+XL_S
B+XL_L
A+XL_L
B+XL_S
z1
c1
z2
b2
z2
c2
z3
z3
c3
z4
z5
b3
c3
c4
z4
c4
c5
y5
c5
c6
z6
y6
y6
c6
intensity
mass
0
50000
100000
150000
200000
250000
500
1000
1500
AAKGNATK+XL_S
AAKGNATK+XL_L
MFEKLEK+XL_S
MFEKLEK+XL_L
A_b_ion
A_y_ion
B_b_ion
B_y_ion
prec
y6++
y5+
b3+
y6+
y7++
y4+
b2+
y2+
b6+
b7+
y7+
y3+
b2+
y4+
b4+
b5+
b6+
y1+
m/z
